# Multiple keratoacanthomas of Ferguson‐Smith type

**DOI:** 10.1002/ccr3.6429

**Published:** 2022-10-11

**Authors:** Noureddine Litaiem, Ferdaous Khammouma, Malek Mrad, Takwa Bacha, Linda Belhadj Kacem, Faten Zeglaoui

**Affiliations:** ^1^ Department of Dermatology Charles Nicolle Hospital Tunis Tunisia; ^2^ Faculty of Medicine of Tunis University of Tunis El Manar Tunis Tunisia; ^3^ Department of Pathology Charles Nicolle Hospital Tunis Tunisia

**Keywords:** dermoscopy, multiple keratoacanthomas of Ferguson‐Smith type, multiple self‐healing squamous epitheliomas, squamous cell carcinoma

## Abstract

We report a case of a 41‐year‐old male patient with no family history, presented with extensive multiple keratoacanthomas with disfiguring scars. The diagnosis of a sporadic form of Ferguson‐Smith syndrome was made. Treatment with acitretin showed a marked response. Recognizing this syndrome is crucial. Early treatment helps avoid scar formation.

## Graphical abstract

Multiple keratoacanthomas of Ferguson‐Smith type is an autosomal dominant condition. Sporadic forms are possible. Multiple self‐healing kératoacanthomas in young adults are suggestive of the diagnosis. The linear distribution of lesions in our patient suggests mosaicism. Continuous treatment with acitretin induces long‐lasting clearance.

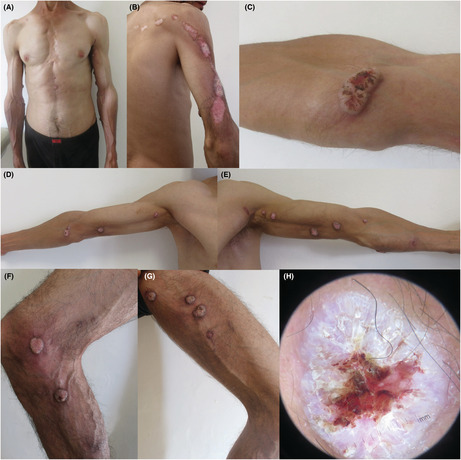

## 1 INTRODUCTION

Kératoacanthoma (KA) is a common cutaneous neoplasm, probably derived from hair follicle cells.^1^, ^2^ KA is a keratin‐plugged, crater‐shaped nodule that arises spontaneously, grows fast, and then typically regresses. It usually presents as a solitary lesion. However, multiple lesions in a sporadic form or an inherited manner are possible.^2^, ^3^ The most common form of multiple KA is Ferguson‐Smith type. We reported herein the first Tunisian case of Ferguson‐Smith type keratoacanthoma that occurred in a 41‐year‐old man, with no family history of this variant.

## 2 CASE REPORT

**FIGURE 1:**
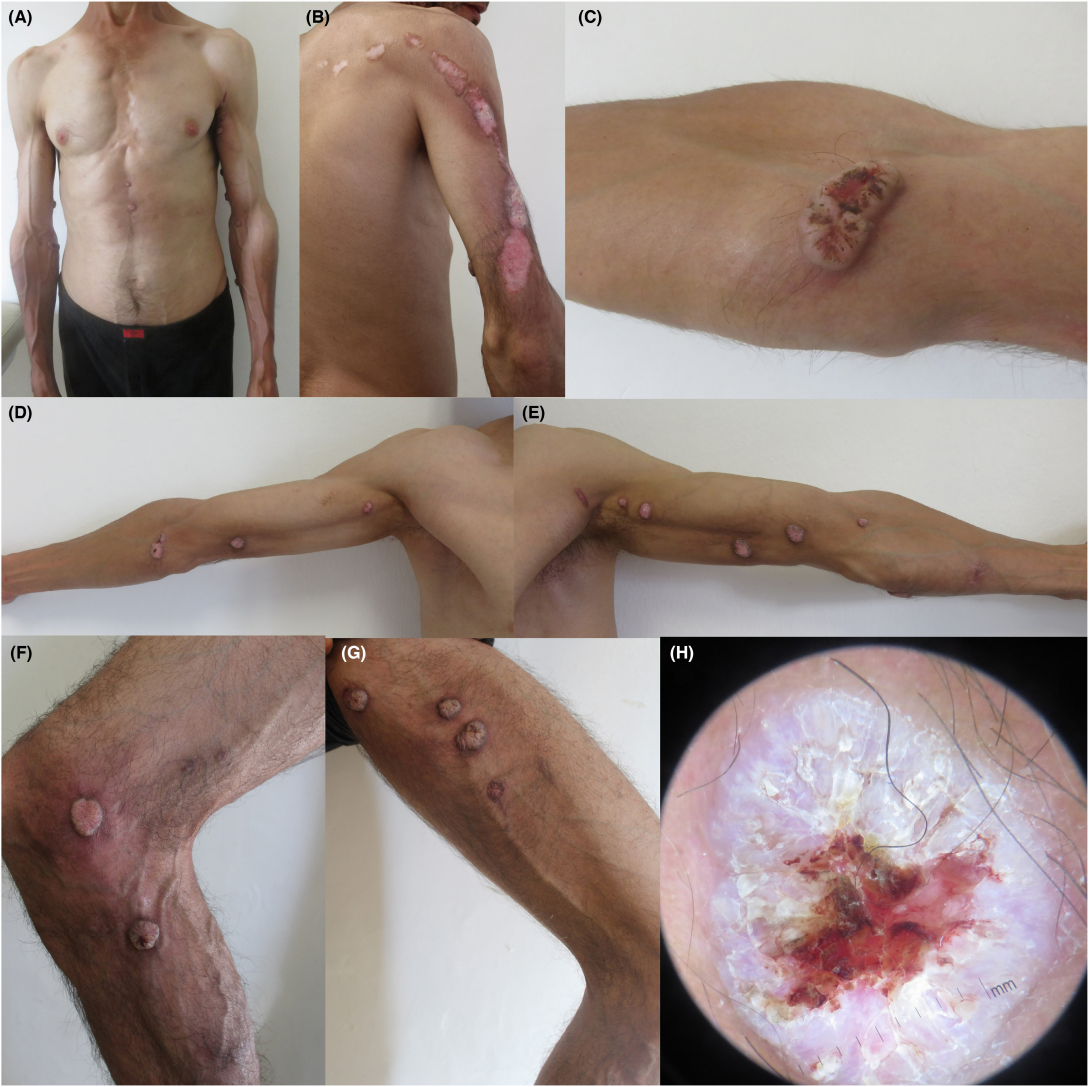
Multiple self‐healing lesions on the trunk (A) and limbs (B–G). Tumors are dome‐shaped with a central keratotic plug, covered with crusts and blood spots (C). Some of the lesions were clustered leaving Blaschko‐linear pitted scars (B). Dermoscopic examination showed a central structureless red‐purple keratotic crater surrounded by a structureless white area with white circles surrounding hair follicles and linear, serpentine, and looped vessels (H).

**FIGURE 2:**
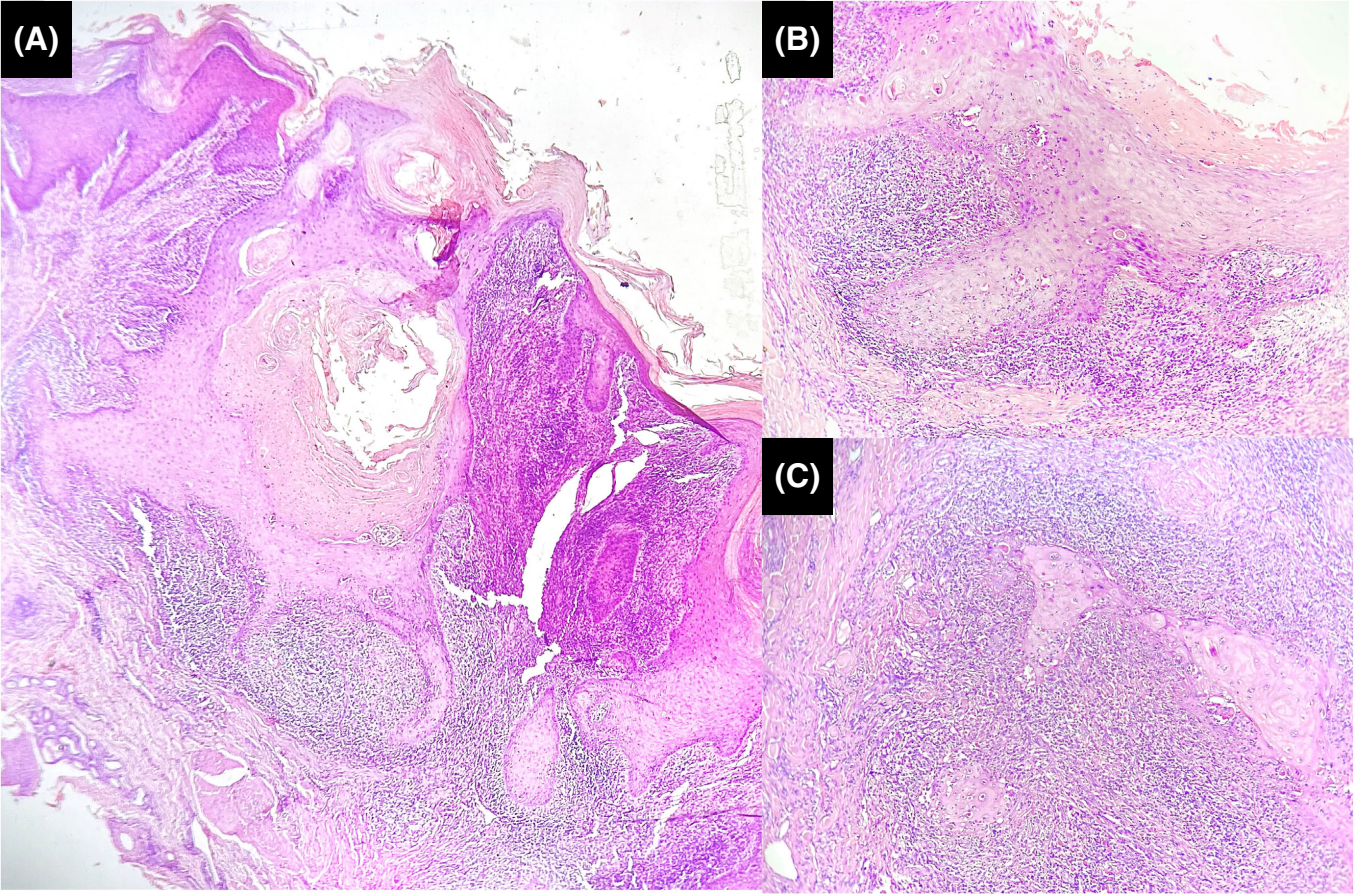
Histopathological examination. (A—Hematoxylin and eosin, ×40) Symmetric cup‐shaped proliferation squamous cells with a central keratinous crater. (b—Hematoxylin and eosin, ×200) the proliferation is made of glassy squamous cells containing abundant eosinophilic and translucent cytoplasm. Mitosis and numerous dyskeratotic cells are visible. (C—Hematoxylin and eosin, ×200) Lymphocytic inflammatory infiltrate surrounding deep lobules of atypical squamous cells.

A 41‐year‐old man, with no medical history, presented with multiple tumors on his forearms and trunk. Lesions appeared in the second decade of his life. Tumors grow slowly and resolve within months, leaving disfiguring scars. Physical examination revealed 23 erythematous, dome‐shaped, cutaneous nodules of 0.5–3 cm diameter, with a central keratotic plug, covered with crusts and blood spots, and located on the trunk and limbs (Figure 1). Some of the lesions were clustered leaving Blaschko‐linear pitted scars. The conjunctiva, palms, soles, genital, and oral mucosa were not affected. Dermoscopic examination showed a central structureless red‐purple keratotic crater surrounded by a structureless white area with white circles surrounding hair follicles and linear, serpentine, and looped vessels (Figure 1). Histopathological examination of an excised tumor revealed a hyperkeratotic acanthotic epidermis with well‐differentiated glassy keratinocytes and basal mitoses, surrounded by a polymorphonuclear inflammatory infiltrate (Figure 2). Based on clinical and histopathological findings, the diagnosis of multiple KA of Ferguson‐Smith type was made. Treatment with oral acitretin at 20 mg/day (approximately 0.3 mg/kg body weight) was started, marked, and rapid response was obtained with no reported side effects.

## 3 DISCUSSION

Clinical forms of multiple KAs include Ferguson‐Smith type, generalized eruptive Grzybowski type, centrifugum marginatum type, multiple persistent non‐familial type, agglomerate type, KAs in Muir‐Torre syndrome, and KAs in xeroderma pigmentosum.^4^

Kératoacanthoma of Ferguson‐Smith type, also known as multiple self‐healing squamous epitheliomas (MSHSE), is the most common form of multiple KAs. It was first described in 1934 in Scottish families. However, sporadic cases have been reported in various countries.^2^, ^4^

Haplotypes for polymorphic markers segregating with Ferguson‐Smith syndrome in non‐Scottish and Scottish families differ, suggesting that Ferguson‐Smith syndrome is not caused by a founder mutation, thus considered now as a digenic/multilocus disease^5^, ^6^ caused by loss of function mutations of *Transforming growth factor, beta receptor I (TGFBR1)* gene interacting with permissive variants at a second linked locus on the long arm of chromosome 9.^7^ Inheritance is autosomal dominant with incomplete penetrance. De novo mutations are possible which may explain the absence of a family history of KA in our patient.

Multiple self‐healing lesions usually appear during childhood, adolescence, or early adulthood. Men and women are equally affected. Although lesions may arise in any part of the skin, KAs are mainly located on the face and extremities. The trunk is rarely affected. Palms and soles are usually spared. Each lesion starts as a reddish macule, becomes papular, and then grows rapidly into an ordinary solitary KA. The number of KAs varies from a few to hundreds.^4^ They evolve and then disappear rapidly within a few months, leaving atrophic and shallower scars and new KAs continue to emerge. The diagnosis of KA of Ferguson‐Smith type can be made based on clinical aspect and distribution of the lesions, histopathological features, age at the onset of the disease, and self‐healing course of the cutaneous tumors. It *can be further confirmed by genetic tests (mutations in the gene TGFBR1)* ^4^

An established association with internal malignancies is reported in cases of multiple KA associated with Muir–Torre syndrome and in other skin conditions such as xeroderma pigmentosum, but not in Ferguson‐Smith syndrome. Only a recently published article on the occurrence of pancreatic cancer in Furguson‐Smith syndrome, yet the authors suggest that this association may be fortuitous.^8^

Retinoids are the first‐line treatment option for Ferguson‐Smith syndrome.^2^ They are associated with a good clinical response. However, they have only a suspensive action. Therefore, a long‐term regimen is necessary to sustain the clinical response. In our case, treatment with a relatively low dose of acitretin was efficient and well tolerated. Limited lesions can be treated with intralesional methotrexate.^9^ Cyclophosphamide was also used with good results in retinoid‐ and methotrexate‐resistant cases of multiple KAs.^10^

## AUTHOR CONTRIBUTIONS

N. Litaiem conceived the idea for the document and contributed to the writing and editing of the manuscript. F. Khammouma contributed to the writing and editing of the manuscript. M. Mrad reviewed and edited the manuscript. The other authors discussed the results by revising critically for important intellectual content and have given final approval of the version to be published. All authors approved the final draft of the manuscript.

## CONFLICT OF INTEREST

The authors declare that they have no conflict of interest or sources of funding for this particular study.

## ETHICAL APPROVAL

Personal data have been respected.

## CONSENT

Written informed consent was obtained from the patient to publish this report in accordance with the journal's patient consent policy.

## Data Availability

Personal data of the patient were respected. No data are available for this submission.

## References

[1] Savage JA , Maize JC . Keratoacanthoma clinical behavior: a systematic review. Am J Dermatopathol. 2014;36(5):8. 10.1097/DAD.0000000000000031 24366198

[2] Kwiek B , Schwartz RA . Keratoacanthoma (KA): an update and review. J Am Acad Dermatol. 2016;74(6):1220‐1233. 26853179 10.1016/j.jaad.2015.11.033

[3] Ko CJ , McNiff JM , Bosenberg M , Choate KA . Keratoacanthoma: clinical and histopathologic features of regression. J Am Acad Dermatol. 2012;67(5):1008‐1012. 22521202 10.1016/j.jaad.2012.02.041

[4] Kato N , Ito K , Kimura K , Shibata M . Ferguson smith type multiple keratoacanthomas and a keratoacanthoma centrifugum marginatum in a woman from Japan. J Am Acad Dermatol. 2003;49(4):741‐746. 14512932 10.1067/s0190-9622(03)00454-7

[5] Ferguson‐Smith MA , Goudie DR . Digenic/multilocus aetiology of multiple self‐healing squamous epithelioma (Ferguson‐Smith disease): TGFBR1 and a second linked locus. Int J Biochem Cell Biol. 2014;53:520‐525. 24747516 10.1016/j.biocel.2014.04.007

[6] D'Alessandro M , Coats SE , Morley SM , et al. Multiple self‐healing squamous epithelioma in different ethnic groups: more than a founder mutation disorder? J Invest Dermatol. 2007;127(10):2336‐2344. 17554363 10.1038/sj.jid.5700914

[7] Goudie D . Multiple self‐healing squamous epithelioma (MSSE): a digenic trait associated with loss of function mutations in TGFBR1 and variants at a second linked locus on the long arm of chromosome 9. Genes. 2020;11(12):E1410. 33256177 10.3390/genes11121410 PMC7760568

[8] Saleh K , Gebre‐Medhin S , Christensen G . Pancreatic cancer occurrence in Ferguson‐Smith syndrome. JAAD Case Rep. 2018;4(6):565‐567. 29998175 10.1016/j.jdcr.2018.05.010 PMC6038265

[9] Moss M , Weber E , Hoverson K , Montemarano AD . Management of keratoacanthoma: 157 tumors treated with surgery or intralesional methotrexate. Dermatol Surg Off Publ Am Soc Dermatol Surg. 2019;45(7):877‐883. 10.1097/DSS.0000000000001739 30608293

[10] Nofal A , Assaf M , Ghonemy S , Nofal E , Yosef A . Generalized eruptive keratoacanthoma: a diagnostic and therapeutic challenge. Int J Dermatol. 2015;54(2):160‐167. 25070745 10.1111/ijd.12308

